# Protocol for a multi-site randomised controlled feasibility study investigating intermittently scanned blood continuous glucose monitoring use for gestational diabetes: the RECOGNISE study

**DOI:** 10.1186/s40814-023-01341-y

**Published:** 2023-07-11

**Authors:** Anna Davies, Erik Lenguerrand, Eleanor Scott, Rebecca Kandiyali, Isabelle Douek, Jane Norman, Abi Loose, Lynn Sawyer, Laura Timlin, Christy Burden

**Affiliations:** 1 Academic Women’s Health Unit, Translational Health Sciences, University of Bristol, Bristol, UK; 2 North Bristol NHS Trust, Bristol, UK; 3 Leeds Institute of Cardiovascular and Metabolic Medicine, University of Leeds, Leeds, UK; 4 Warwick Clinical Trials Unit, Warwick University, Coventry, UK; 5 Somerset Foundation NHS Trust, Somerset, UK; 6 NHS England-South-West, Taunton, UK

**Keywords:** Gestational diabetes, Continuous glucose monitoring, Feasibility study, Large for gestational age

## Abstract

**Background:**

Incidence of gestational diabetes mellitus (GDM) is increasing and is associated with adverse perinatal outcomes including macrosomia, pre-eclampsia, and pre-term delivery. Optimum glycaemic control can reduce these adverse perinatal outcomes. Continuous glucose monitoring (CGM) informs users about interstitial glucose levels allowing early detection of glycaemic excursions and pharmacological or behavioural intervention. Few adequately powered RCTs to evaluate the impact of using CGM in women with GDM on perinatal outcomes have been undertaken.

We aim to establish the feasibility of a multi-site RCT to evaluate the clinical- and cost-effectiveness of an intermittently scanned continuous glucose monitor (isCGM) compared with self-monitored blood glucose (SMBG) in women with GDM for reducing fetal macrosomia and improving maternal and fetal outcomes. We will evaluate recruitment and retention rates, adherence to device requirements, adequacy of data capture and acceptability of trial design and isCGM devices.

**Methods:**

Open-label multicentre randomised controlled feasibility trial. Inclusion criteria: pregnant women, singleton pregnancy, recent diagnosis of GDM (within 14 days of commencing medication, up to 34 weeks gestation) prescribed metformin and/or insulin. Women will be consecutively recruited and randomised to isCGM (FreestyleLibre2) or SMBG. At every antenatal visit, glucose measurements will be evaluated. The SMBG group will use blinded isCGM for 14 days at baseline (~ 12–32 weeks) and ~ 34–36 weeks. The primary outcome is the recruitment rate and absolute number of women participating. Clinical assessments of maternal and fetal/infant health will be undertaken at baseline, birth, up to ~ 13 weeks post-natal. Psychological, behavioural and health economic measures will be assessed at baseline and ~ 34–36 weeks gestation. Qualitative interviews will be undertaken with study decliners, participants, and professionals to explore trial acceptability, of using isCGM and SMBG.

**Discussion:**

GDM can be associated with adverse pregnancy outcomes. isCGM could offer a timely, easy-to-engage-with intervention, to improve glycaemic control, potentially reducing adverse pregnancy, birth and long-term health outcomes for mother and child. This study will determine the feasibility of conducting a large-scale multisite RCT of isCGM in women with GDM.

**Trial registration:**

This study has been registered with the ISRCTN (reference: ISRCTN42125256, Date registered: 07/11/2022).

**Supplementary Information:**

The online version contains supplementary material available at 10.1186/s40814-023-01341-y.

## Background

Gestational diabetes mellitus (GDM) is glucose intolerance with onset during pregnancy, typically resolving after birth [1, 2] GDM affects 5–10% of UK (United Kingdom) pregnancies (35,000–70,000 women yearly) [2, 3]. Incidence estimates vary due to different screening methods, but is increasing rapidly due to higher rates of obesity and pregnancies in older women [4, 5].

Women diagnosed with GDM are primarily advised about lifestyle changes. If these are ineffective in controlling blood glucose, they are offered medications such as metformin and/or insulin, depending on local or national guidance [3]. Recommended current standard care for women with GDM in the UK is self-monitoring of capillary blood glucose (SMBG) four to seven times daily using ‘finger-prick’ testing [3]. Barriers to SMBG include stigma of public testing, anxiety, pain and inconvenience [6]. Moreover, the glucose variability and transient glucose excursions from target blood glucose ranges that may be experienced during pregnancy are not easily detectable using SMBG [6, 7].

High levels of glycaemia in women with GDM are associated with risk of adverse pregnancy and birth outcomes for mother and baby. Recent studies [8–10] and a meta-analysis [11] have investigated the association between GDM and adverse pregnancy outcomes. In a meta-analysis of 156 studies of over 7 million pregnancies, where data was examined for women who did not use insulin, women with GDM compared with those without had increased risk of caesarean section, pre-term delivery, low 1 min Apgar score, fetal macrosomia, and large for gestational age (LGA) infant. In women with GDM who were prescribed insulin (in whom lifestyle changes may not have achieved target glucose levels) there was increased risk of having an LGA infant, an infant with respiratory distress syndrome, jaundice, or requiring neonatal intensive care (NICU) admission compared with women without GDM [11]. Improved glycaemic control can reduce these complications [11].

High overnight glucose levels in women with GDM are associated with fetal macrosomia, which go undetected by SMBG [12]. To address the shortcomings of SMBG, continuous glucose monitoring devices (CGMs) have been developed, which measure interstitial fluid glucose levels using a small subcutaneous sensor. These provide more frequent measurements and a more detailed picture of glucose levels over 24 h, identifying glucose fluctuations. They can immediately alert women to excursions (hypo/hyperglycaemia) using alarms [13]. The intermittently scanned Continuous Glucose Monitor-isCGM (or “Flash” device) provides continuous glucose data if the patient swipes their arm sensor at least every 8 h with a smartphone/reader. CGM data enable better clinical decision-making about medication and behavioural modifications, (diet, physical activity, glucose monitoring, and medication adherence) to help control glucose levels. The healthcare team can also remotely monitor women, reducing burden on services and users. CGM could also provide better oversight in those who do not reach advised glucose targets and potentially in those who do not speak the same language as healthcare providers.

Systematic review data indicates that CGM devices improve clinical outcomes in non-pregnant adults with diabetes, reducing HbA1C and time in hypo/hyperglycemia [14, 15]. In pregnant women with type 1 diabetes mellitus, the CONCEPTT trial demonstrated less glycaemic variability, reduction in fetal macrosomia and NICU admissions in those using CGM devices compared with SMBG [16]. The use of CGM devices has become commonplace outside pregnancy, and is recommended by NICE for pregnant women with type 1 diabetes mellitus [17]. However, systematic reviews indicate that little research has investigated CGM use in women with GDM; a Cochrane review identified two low-quality RCTs comparing CGM and SMBG in women with GDM, and found no difference in outcomes for mother or baby, including caesarean rate, fetal macrosomia, neonatal hypoglycaemia, and perinatal death [18]. The most recent systematic review and meta-analysis of six RCTs investigating the effect of CGM compared with SMBG identified that CGM use in GDM was associated with reduced HbA1c levels, lower gestational weight gain, and lower birth weight, but no other outcomes measured including mean fasting or postprandial blood glucose, hypertensive disorders, fetal macrosomia/LGA, or neonatal hypoglycaemia [19].

Together, these data indicate limited high-quality evidence examining the use of CGM devices in women with GDM in pregnancy. The studies described all have major limitations: extremely small sample sizes, limiting their statistical power to detect changes in clinical outcomes; short-term device usage rather than throughout pregnancy; use of less accurate, now outdated, devices that did not always provide real-time data, and sub-optimal adherence to device requirements [18, 20, 21]. No RCT has targeted women who are prescribed metformin or insulin who have not reached glycaemic targets with lifestyle interventions, and in whom the most benefit is likely to be achieved. It will result in having additional data to inform care if they have not adhered to regular SMBG and a clearer picture for clinicians of where within the 24-h window problems may lie, to advise lifestyle and medication changes. Furthermore, no studies report investigating the acceptability of the intervention, particularly in relation to different sociodemographic groups or underserved women, in whom increased risk of adverse pregnancy outcomes has been identified [22].

To date, no RCT has been conducted to establish the effectiveness, safety and cost-effectiveness of isCGM devices in women with GDM, despite the potential substantial health improvement for both mother and baby. Feasibility work is needed to determine whether a high-quality multi-site RCT can be conducted, to assess whether women will agree to participate in an RCT, acceptability of randomisation and data collection processes, and whether adequate data capture can be achieved to inform efficacy and health economic analyses. In addition, it is important to understand adherence to the device requirements and acceptability of isCGM and using masked devices within a control arm.

The RECOGNISE (taRgeted intermittEnt gluCose mOnitoring for the management of GestatioNal dIabeteS mEllitus) study aims to establish the feasibility of an RCT to evaluate the clinical- and cost-effectiveness of CGM devices (an intermittently scanned-isCGM device), compared with SMBG, for reducing fetal macrosomia, and improving pregnancy and health outcomes in women diagnosed with GDM who have been prescribed medication for management.

Research questions:

1. Will women agree to participate in an RCT comparing isCGM and SMBG, and is randomisation acceptable?
2. Do women adhere to the requirements of the isCGM device?
3. Can we collect the data necessary to understand clinical effectiveness, experience and health economic outcomes (completeness of self-report data, glycaemic control monitoring data, and extraction from medical records)?
4. Is isCGM acceptable to women with GDM? Does this vary by socio-demographic factors?
5. Is isCGM acceptable to healthcare professionals who care for women with GDM?
6. Do women and healthcare professionals find glucose readings from isCGM devices useful to inform treatment modification and management, and to optimise glycaemic control?

## Method

**Fig. 1:**
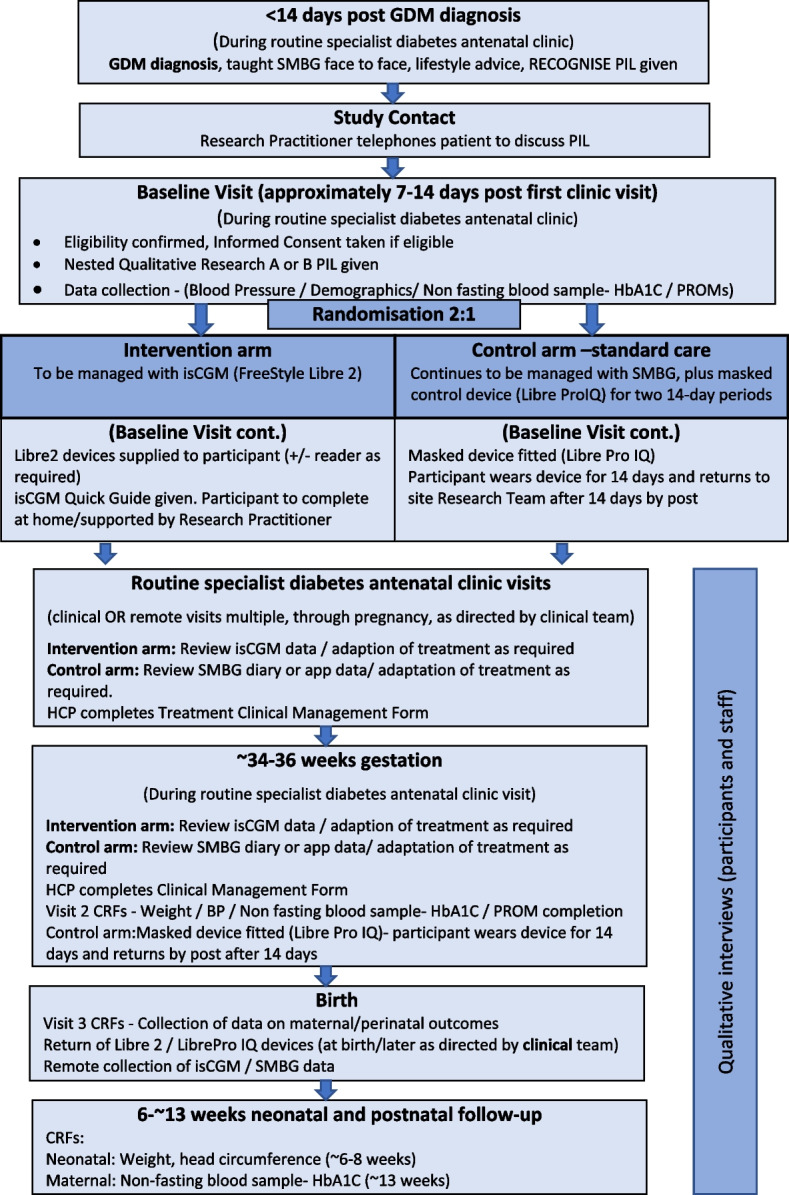
Study flow diagram

**Fig. 2:**
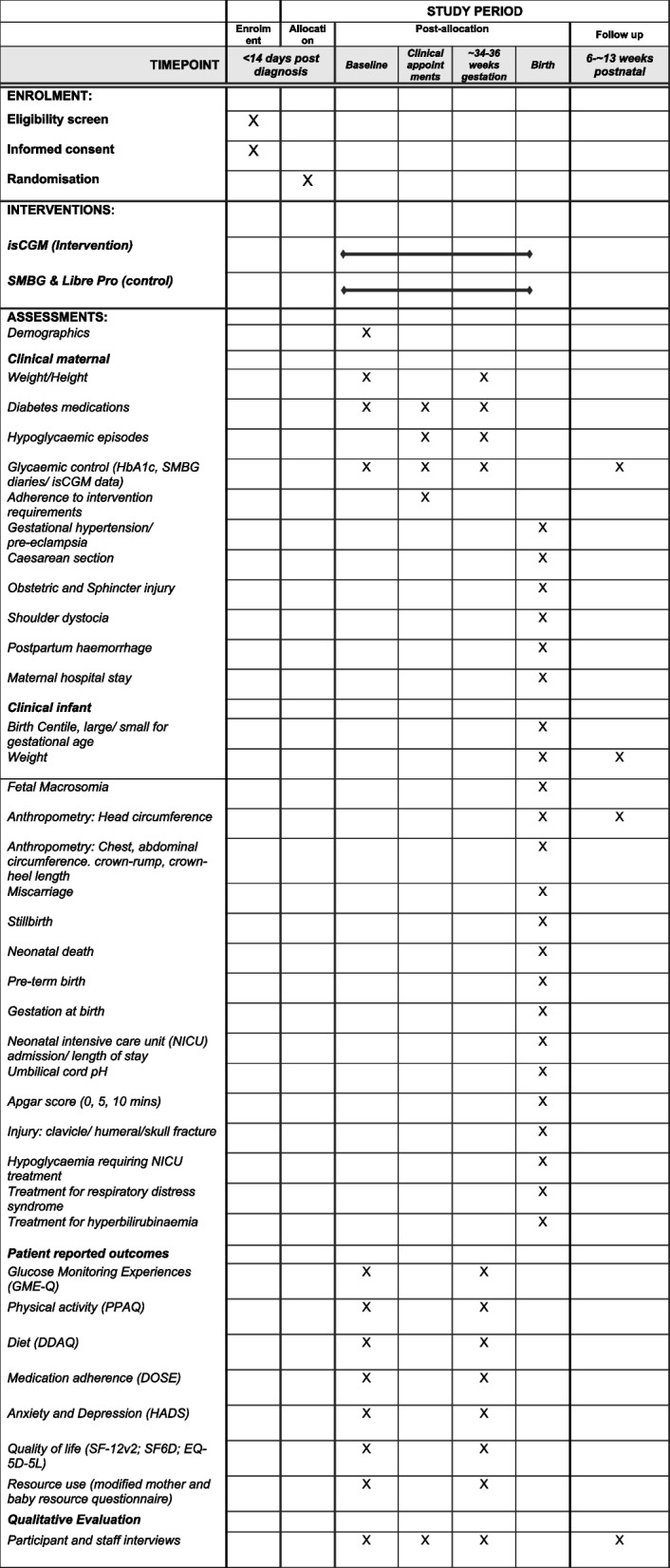
SPIRIT figure

This protocol adheres to the SPIRIT framework for reporting of study protocols [23]. Figure 1 describes the study flow and Fig. 2 provides the SPIRIT figure.

### Study design and setting

This study is designed as a multi-centre, open, randomised controlled feasibility study with embedded qualitative research, conducted in two UK maternity units: North Bristol NHS Trust (NBT) and Somerset NHS Foundation Trust (SFT). The feasibility study is sponsored by North Bristol Trust.

### Participants

Women who have diagnosed GDM in their current pregnancy according to NICE criteria (2-h 75 g oral glucost tolerance test [OGTT]), who have not established target glycaemic control using lifestyle modification alone will be recruited up to 34 + 0 weeks of pregnancy. Table 1 indicates inclusion and exclusion criteria for the study. We will use the National Institute for Health and Care Research (NIHR) INCLUDE Framework to achieve representation of underserved (including black and minority ethnicity) women [24].

**Table 1:** Inclusion and exclusion criteria

Inclusion	Exclusion
• Aged 16–55 • GDM diagnosed at any gestation (typically > 12 weeks) per NICE guidelines: oral glucose tolerance test demonstrating a fasting plasma glucose level of 5.6 mmol/litre or above or a 2-h plasma glucose level of 7.8 mmol/l or above [3] • Has commenced medication (metformin ≥ 500 mg/day) and/or insulin) within the previous 14 days • ≤ 34 + 0 weeks of gestation • Primiparous or multiparous • Singleton pregnancy • Not met NICE glucose targets with lifestyle modification of fasting glucose below 5.3 mmol/l, 1 h after meals below 7.8 mmol/l OR 2 h after meals below 6.4 mmol/l [3] • Able to give informed consent to participate	• Aged < 16 or > 55 • Not prescribed insulin or at least 500 mg/day metformin, OR commenced medication > 14 days ago • Met NICE glucose targets with lifestyle modification • > 34 + 0 weeks gestation • Chronic kidney disease • Psychiatric inpatient treatment, • History of bariatric surgery or other surgeries that induce malabsorption • Long-term use (> 2 weeks) of systemic steroids within 2 weeks prior to enrolment • Multiple pregnancy • Unable to give informed consent to participate

### Screening and consent

Women will be informed of the study by their healthcare professional (HCP); obstetrician, endocrinologist, diabetes specialist nurse, or specialist midwife in their first specialist diabetes antenatal clinic appointment where they are referred after diagnosis of GDM. Interested patients will be given a Participant Information Leaflet (PIL). PILs will be translated into four languages, targeting the groups of women who do not speak English as a first language most frequently seen in our GDM clinics: Arabic, Bangladeshi, Polish, and Romanian. All women will also have access to a recruitment video (with preferred language voiceover/subtitles). Patients will be followed up with a telephone call by a Research Practitioner to discuss the study prior to their next appointment, with verbal translation used as needed. At their next specialist diabetes antenatal clinic appointment, the clinician will identify women meeting the above inclusion criteria—those who have not met the glucose control targets and therefore are prescribed metformin (≥ 500 mg/day) and/or insulin. They will be invited to participate, have an opportunity to ask questions and have written informed consent obtained by a Research Practitioner.

### Sample size

#### Quantitative sample size

There are approximately 6000 and 3000 births/year at NBT and SFT, respectively. At NBT 440 patients are diagnosed with GDM each year (180 and 90 women per year on insulin or at least 500 mg/day of metformin will be seen in clinics after exclusions, in NBT and SFT respectively). With a 60% participation rate of those eligible women, we could recruit 108 per year at NBT and 54 per year at SFT. A 12-month recruitment period at both sites will be sufficient to meet our target, allowing for unforeseen recruitment delays. We will be able to estimate a participation rate of 60% to within 95% confidence interval of 52% to 68%.

#### Qualitative sample size

The qualitative sample size estimation is pragmatic, accounting for study sample size (*n* = 60) and information power [25], whereby sample size is determined by the research aims (narrow/broad), theoretical underpinnings (whether theory informs the topic guide), and dialogue quality (researcher topic-related knowledge), and within versus cross-case analysis. An experienced researcher with diabetes-specific knowledge will carry out this work, which has focused aims. Behavioural science frameworks will inform the topic guides and a cross-case analyses will be conducted. Recruitment of up to 20 intervention group (12–14 NBT; 6–8 SFT), and 10 control group participants (6–7 NBT; 3–4 SFT) represents approximately 50% of study participants, balancing proportion of expected recruitment at each site. A further 6–8 women declining participation (4–5 NBT, 2–3 SFT), and up to 10 healthcare professionals (HCPs) across sites (6–7 NBT, 3–4 SFT) is considered an appropriate sample size to answer the questions addressed.

### Randomisation and masking

Participants and HCPs/those collecting outcome data cannot be masked to allocation. Women will be randomised in a 2:1 ratio to isCGM device or SMBG. Women will be allocated to their study group using a minimisation strategy which will account for site (NBT/SFT), Insulin (yes/no), and gestation at diagnosis (early (< 24 + 0 weeks)/late(≥ 24 weeks)), using the Minirand command in R [26]. The allocation will not be revealed until sufficient data to identify the participant has been entered to ensure allocation concealment. Randomisation will be requested by the member of staff responsible for recruitment for the trial from a member of staff who is independent of the trial. A 2:1 (isCGM or SMBG) allocation ratio was chosen for the feasibility study to reduce the required sample size while increasing the efficiency of the study, and without compromising the study main objectives.

### Intervention/procedures

Women will be assigned to: Intervention: monitoring their glucose levels using an isCGM device (Freestyle Libre2) and smartphone/reader; or Control: standard care using SMBG.

#### Intervention group

The isCGM device-FreeStyle Libre2 sensor (Abbott Diabetes Care, Alameda, CA, USA) is a second-generation Libre device. It consists of an integrated transmitter and sensor, packaged with a disposable self-insertion kit. The sensor reads to a mobile phone app (or a reader for people without a compatible phone). The device measures interstitial glucose through the small sensor inserted on the back of the upper arm to provide real-time glucose readings. Women will be asked to swipe their sensor a minimum of five times a day at the time of waking, 1 h post-meals and pre-bedtime to obtain continuous data. The device has an in-built algorithm, to reduce the time-lag associated with measuring interstitial glucose, thereby providing nearer real-time results. The FreeStyle Libre2 sensor we intend to use has customisable alarms, allowing the women to be alerted to out of target glucose levels. For women with a smartphone, the device app will be installed. A reader with identical data output will be provided for those without, so as not to exclude those unable to afford/choosing not to have a smartphone. Data from mobile devices are transmitted in real time to LibreView, a cloud-based system accessible to both patients and HCPs. Device data will be available to women (via their smartphones/reader) and their HCPs using the online interface (at each visit) and used to modify treatment and behaviour. The monitor provides the user with information about current glucose levels and when glucose levels change. All intervention group participants will be trained to wear and use the system and how to exchange a sensor using a brief ‘getting started’ leaflet, and access to manufacturer online tutorial videos available to all Freestyle Libre2 users [27].

#### Control group

The control group will use SMBG and a paper or smart phone application (app) diary (GDm-Health; Sensyne Health [28]) which is recommended by NICE [29], The control group will perform SMBG testing with a blood glucose meter, including testing supplies (as per current standard care at both sites). They will be asked to perform SMBG routinely used for women with GDM, that is, at least four capillary blood glucose values daily, including measurements in a fasting state as well as 1 h after starting each meal (also additional tests pre meals if on insulin). Women will be asked to keep a logbook of their glucose values, either as a paper diary or using the GDM Health app for review by HCPs at each visit per routine clinical practice at each maternity Unit. Women using GDM Health will be sent messages per standard local practices.

They will also be given a masked isCGM device (FreeStyle Libre Pro) to wear for 14 days at two time points: baseline and ~ 34–36 weeks gestation. Women will be advised how to wear the masked device but will not be asked to download the app or take readings, such that glucose readings will not be available for women or HCPs.

### Both groups

All women diagnosed with GDM in pregnancy receive standard lifestyle advice on their diet and exercise regimes and are shown how to undertake SMBG (fasting and 1 h post-meal) by an HCP at their initial appointment at each hospital as per current Maternity Unit practice. Women are followed up and seen for the remainder of the pregnancy in the specialist diabetes antenatal clinic by obstetricians, endocrinologists, midwives, specialist dietitians, and diabetes specialist nurses. They will be seen in antenatal clinic visits monthly as per current care pathways, ensuring points of contact are comparable across groups. Both groups will be advised to stay in target range glucose levels in accordance with national NICE recommendations (fasting < 5.3 mmol/l; 1 h after meals < 7.8 mmol/l [3]. Treatment will be titrated up by the clinical care team using metformin and/or insulin if glucose levels are out of range more than twice at a time point and no further dietary intervention is possible.

### Outcomes

The primary outcome for this feasibility study is the recruitment rate and the absolute number of women participating in the study. Recruitment outcomes will include: number of women invited to study, number of women meeting eligibility criteria, number of women recruited, and follow-up and dropout rates (attrition). Reasons for declining participation will be explored in the embedded qualitative study.

Adverse events are defined as any untoward medical occurrence in a randomised patient regardless of its causal relationship to study treatment. Provision has been made for actively recording adverse events, which are expected, for example, pain or bleeding during the device application. All adverse events that occur during the study period will be documented on a designated data form. Adverse events will be recorded and reported in accordance with the Sponsor’s Safety Reporting SOP.

### Data completeness

We will collect clinical, psychosocial, behavioural, and health economic data to estimate the required sample size for a full-scale RCT and to inform us about data completeness. Clinical outcomes have been selected with reference to the Core Outcome Set for studies investigating GDM prevention and treatment [30]. Antenatal, birth and post-natal visit data will be recorded from the woman's and infant’s medical records and using a case report form (CRF) at birth. Outcomes, tools used and timepoints for measurement are presented in Table 2. Data completeness will be assessed as number of randomised women completing questionnaires (whole questionnaire).

**Table 2:** Outcomes measured

Clinical fetal/neonatal outcomes from clinical record (birth, 6–8 weeks post-natal where indicated)
• Birth centile including large and small for gestational age (LGA; SGA); Intergrowth birth centile chart. LGA > 90th centile SGA < 10th centile
• Birth weight (g) • Fetal macrosomia > 4 kg (yes/no)
• Anthropometry: Head, chest, abdominal circumference; crown-rump length, crown-heel length (cm, birth); and weight (g) and head circumference (cm) (birth and 6–8 weeks post-natal)
• Miscarriage before 23 + 6 gestation (yes/no) (as arising)
• Stillbirth after 24 + 0 gestation (yes/no) (as arising)
• Neonatal death < 28 days of life (yes/no) (as arising)
• Preterm birth < 37 weeks gestation at birth (yes/no)
• Gestation at birth (weeks)
• Neonatal intensive care unit (NICU) admission (yes/no) and length of stay in days
• Umbilical cord pH
• Apgar score at 0, 5, 10 min [31]
• Injury: clavicle, humeral, skull fracture (yes/no)
• Hypoglycaemia requiring NICU treatment (yes/no)
• Treatment for Respiratory distress syndrome (yes/no)
• Treatment for hyperbilirubinaemia (yes/no)
Clinical maternal outcomes from clinical record (timepoints as indicated)
• Weight (kg) (booking, 34–36 weeks)
• Height (cm) (booking, 34–36 weeks)
• Diabetes medications prescribed (type, quantity) (each appointment)
• Hypoglycaemic episodes (if on insulin) (number/frequency) (each appointment)
• Other adverse events (e.g. skin irritation) (each appointment)
• Glycaemic control: • HbA1c baseline (34–36 weeks, 6– ~ 13 weeks post-natal) • SMBG daily diaries—fasting and post-meal glucose (each antenatal appointment; SMBG only) • Continuous glucose data—% time in target range (3.5–7.8 mmol/l), area under curve, % time in hypo- and hyperglycaemia, standard deviation and amplitude of glycaemic excursions, (baseline, 34–36 weeks)
• Gestational hypertension/pre-eclampsia per NICE diagnostic criteria (yes/no) [32] (at birth)
• Induction of labour (yes/no) (at birth)
• Caesarean birth (pre-labour or intrapartum)/instrumental birth/vaginal birth (yes/no) (at birth)
• Obstetric and sphincter injury (OASI) (yes/no) (at birth)
• Shoulder dystocia (yes/no) (at birth) • Postpartum haemorrhage (yes/no) (at birth)
• Maternal hospital stay (days) (at birth)
Psychosocial, behavioural, and health economic (patient reported) baseline and ~ 34–36 weeks gestation
• Glucose monitoring experiences questionnaire (GME-Q) [33]
• Diabetes self-care behaviours (diet, physical activity, medication adherence, glucose monitoring) • Pregnancy Physical Activity Questionnaire (PPAQ) [34] • UK Diabetes and Diet Questionnaire (UKDDQ) [35] • Voil’s DOSE medication non-adherence measure [36]
• Anxiety and Depression: Hospital Anxiety and Depression Scale (HADS) [37]
• Quality of life: SF-12v2 [38]; SF-6D [39]; EQ-5D-5L [40];
• Patient reported resource use questionnaire Baseline/Visit 2 Mother Resource Questionnaire-study specific. Modified with public-patient involvement from the Mother and Baby Resource Questionnaire, with permission from Warwick Clinical Trials Unit

### Adherence to intervention requirements

To prevent missing data women in the intervention group will be advised by HCPs to swipe the isCGM device at least five times daily (fasting, 3 × post-prandial, pre-bedtime). International consensus on the use of continuous glucose monitors describes 70–80% of data over a 7–14 day period is required for data sufficiency [41]. This will be assessed at each antenatal routine visit.

### Health economic data collection

The economic feasibility will focus on data collection to inform the economic evaluation to be done alongside the future definitive RCT. The future evaluation is likely to present results in cost/QALY terms reporting within trial and lifetime horizons and from an NHS/personal social services (PSS) perspective. The economic feasibility work will focus on establishing the appropriate methods for collecting the outcomes—both costs and utilities—which will be of interest for the future evaluation. We will record the number of prescribed SMBG testing strips/monitors, continuous glucose monitoring devices/readers from women's secondary care notes. We will extract from notes and other hospital resources relating to GDM, which may include additional laboratory tests ordered and the aforementioned antenatal, pregnancy and neonatal complications (see “Clinical outcomes” section) including length of stay. Use of specific primary and personal social services will be assessed using a bespoke patient reported resource use questionnaires at baseline and ~ 34–36 weeks developed with public patient representative involvement. We will ask patients to complete both the SF-12v2 (applying the SF-6D algorithm to obtain health state utilities) [38] and EQ-5D-5L [40] at baseline and ~ 34–36 weeks gestation to guide the selection of a utility-based measure for the full study. Recent publications in the target or similar conditions have not explicitly broached the selection of outcome measures [42], so we would like to use the opportunity to examine completeness and floor/ceiling effects.

While the focus for the feasibility is on testing and refining health economic methods of data collection, we anticipate that we might be able to present some very preliminary cost analyses to inform the economic case for the future study. Such an analysis might consider the circumstances where isCGM is likely cost-neutral or saving and will be exploratory.

### Adverse event monitoring

Adverse events will be recorded as part of the case report form and collected from the patient record at each scheduled participant visit (or sooner if initiated by the participant between visits/if notified by the clinical team). Adverse events will be reviewed relating to seriousness (defined as: results in death, is life-threatening, requires hospitalization, results in persistent or significant disability or incapacity, consists of a congenital abnormality or birth defect, is otherwise considered medically significant by the investigator). Serious adverse events will be notified to the CI/PI (or delegated study clinician) and reviewed as to relatedness to study/procedures and expectedness. Serious adverse events related to procedures or intervention, and which are unexpected will be reported to the Research Ethics Committee. In the unlikely event of harm resulting from participation in the trial, NHS indemnity applies to any patient in the NHS.

### Patient retention methods

Every reasonable effort will be made to collect data from all participants for the entire course of the study. Sponsor approved processes for retaining participants will be followed within the study. These include contact via email or phone call on up to three occasions at each data collection point to enable capture of participant data and text reminders to bring their CGM monitor for data download/App or paper diary to each clinical appointment.

### Statistical analysis

The participant’s baseline characteristics will be tabulated using mean, standard deviation or median (interquartile ranges) depending on their distribution, and percentages and frequencies for categorical data. We will report on frequency and percentages for the feasibility outcomes: recruitment rate, withdrawal rate, intervention compliance, data completion. Safety outcomes will also be presented as counts and percentages of occurrences. We will report these feasibility study outcomes by randomisation groups to identify challenges specific to any of the groups. There will be no statistical comparison of these measures and their potential confidence intervals by randomisation group as this is a feasibility study.

The health economic analysis will be limited to descriptive statistics (i.e. frequency and percentages) focusing on completeness of health economic outcome measures and resource items. Any analysis of incremental costs will be considered exploratory and will outline the assumptions and uncertainty (standard errors) for each scenario.

### Embedded qualitative research

We will conduct two qualitative studies. We will interview women invited to participate in the feasibility study, to explore experiences of participation, reasons for declining, and acceptability and experiences of the intervention investigated. We will also conduct interviews with healthcare professionals (HCPs) and research staff involved in delivering the feasibility study and clinical care to explore experiences of study recruitment and acceptability of the isCGM devices when delivering care.

#### Qualitative study 1: interviews with women invited to participate in the feasibility study

##### Participants

All women meeting the eligibility criteria for the feasibility study (see Table 1) will be invited to take part. We will screen women who express interest in participating, and purposively sample them using maximum variation sampling for diversity relating to age, parity, previous GDM, socio-economic status, and ethnicity.

Three groups of women will be recruited:

1. Intervention group participants (*n* = 20; 12–14 NBT; 6–8 SFT)
2. Control group participants (*n* = 10; 6–7 NBT, 3–4 SFT)
3. Women declining participation in the feasibility study (*n* < 8; 4–5NBT, 2–3 SFT)

Groups 1 and 2 will be invited to participate during the intervention period or after follow-up completion (approximately 13 weeks post-natal). We will aim to recruit up to four women who have withdrawn or not completed the study to enable representation of their views. Participants will receive a study information sheet, be given time to consider taking part, and written, informed consent to participate will be sought by the researcher. Interviews will take place face to face, or remotely by telephone or video conferencing as preferred by the participant.

Women eligible for the feasibility study but declining participation (group 3) will be invited to participate within 1 week of their decision to decline. A study information sheet will be provided (including translation into Arabic, Bangladeshi, Polish, Romanian) and participants will be given time to consider participation. Interviews will be conducted by video conferencing or telephone. Audio-recorded, informed consent will be sought by the researcher with a record of the verbally completed consent form emailed to participants.

Interviews will be conducted by an experienced qualitative researcher, with the assistance of a translator where the participant wishes to participate in a language other than English. Interviews will be audio recorded and transcribed verbatim. Participants will be reimbursed for their time and expenses incurred.

##### Topic guide

Groups 1 and 2: A semi-structured topic guide will be developed with the Patient Partner Group (PPG), to explore women’s experiences relating to 1. Recruitment to and participation in the study, and 2. Acceptability of the isIGM devices (unmasked and masked). Acceptability will be investigated using the Theoretical Framework of Acceptability [43] (TFA) to explore affective attitudes, burden, ethicality, intervention coherence, opportunity-costs, perceived effectiveness, and self-efficacy. Interviews will last up to 1 h.

Group 3: Interviews will last 10–15 min to minimise burden for participants who have declined participation. Questions and prompts will explore reasons for declining including research participation concerns, isCGM devices, trial group requirements, and data collection.

#### Qualitative study 2: qualitative study with health care professionals

Methodology is adapted from the Qualitative Research Integrated in Trials (QuInteT) framework [44].

##### Participants

Up to 10 HCPs (6–7NBT, 3–4 SFT) will be purposively recruited during and following study completion to ensure representation across roles/timepoints. Some HCPs are expected to both recruit and deliver isCGM-related care. Eligible participants will be midwives, obstetricians, endocrinologists, dieticians, diabetes specialist nurses, diabetes specialist midwives and research midwives if they are involved in at least one of the following activities: assessing participant eligibility, introducing the study, recruiting, consenting and on-boarding women, and delivering clinical isCGM-related care.

HCPs will be invited via email from the study lead or direct approach from the research team. They will be given a participant information leaflet and time to consider taking part. Written, informed consent to participate will be sought. Interviews will take place face-to-face, by telephone or online via videoconferencing software per participant preference. Interviews will be audio-recorded and transcribed verbatim.

##### Topic guide

A semi-structured topic guide will be developed with the PPG and Study Steering Committee (SSC), to explore perceptions of the study protocol, recruitment barriers and pathways. Acceptability of isCGM devices for supporting care in women with GDM will be explored using the TFA [43], as described above.

### Analyses (qualitative studies 1 and 2)

All analyses will be carried out by the qualitative lead. Transcripts will be uploaded to NVivo for analysis. Concurrent analyses will be carried out, to enable topic guide refinement and prevent unnecessary recruitment. Deductive and inductive thematic analyses will be conducted to explore questions around participation and intervention acceptability, with the opportunity to identify unanticipated themes [45]. An independent researcher will review up to four transcripts to co-identify themes and will code a further 2–4 transcripts using the agreed themes to ensure analytic validity. Findings will be reported using the COnsolidated criteria for REporting Qualitative research (COREQ) checklist [46].

### Data management strategy

The database and randomisation system will be designed to protect patient information in line with (i) the Data Protection Act 1998 until 24 May 2018, and (ii) the General Data Protection Regulation, as from time to time amended from 25 May 2018. Consent forms will be completed per patient preference, either online using REDCap, a secure web-based software platform designed to support data capture for research studies, or on paper copies. Patient reported outcome measures will be completed directly into the REDCap study database, or on paper questionnaires during study visits or at home within 5 days of the visit.

Research practitioners will systematically collate clinical data from the patient records (electronic or handheld notes, as per local provision) and input directly to the bespoke study database (REDCap). Where paper completion of case report forms or patient reported outcome measures has been used in place of direct electronic capture, data will be transcribed to the study database in a timely manner (usually within 24 h of their collection). Paper copies of data will be stored in a locked filing cabinet at NBT/SFT.

Onsite and/or remote monitoring of study data will be conducted after each study visit of the first participant to reach each milestone (baseline, 34–36 weeks, birth, 6–13 weeks post-natal). Further monitoring visits will follow a proportionate risk monitoring plan. At the end of the study a final monitoring visit will take place after all women have been recruited and have completed the post-natal visit. This visit will be used to review the completeness and filing/archiving arrangements for consent forms, delegation and accountability logs, site files, and training logs. A minimum 10% sample of completed CRFs will undergo source data verification and a quality control check of data entered into the study database.

### Participant withdrawal

Participants are free to withdraw from the study at any point. This will not affect their ongoing care. If a woman withdraws from the study, she will be managed by the clinical team as per routine care guidelines. Participants recruited into the intervention arm will be returned to standard care SMBG. Participants using either type of monitoring will be advised to monitor blood glucose using SMBG until birth, but no further data will be collected for research purposes. A record will be kept of participants who withdraw along with their reasons for doing so, where given, on a study withdrawal form. This will allow the participant to specify whether they will allow further collection of data from their medical record. Women participating in the qualitative arm will be allowed to withdraw consent for use of their data up to the point of anonymised transcription.

Participants who experience miscarriage or stillbirth will be withdrawn from further patient-reported data collection automatically (without the need for completion of the study withdrawal form). Data derived from the clinical record up to and including the end of the pregnancy will be included in the study unless the participant wishes to withdraw from collection of this data via completion of the withdrawal form.

### Success criteria

Success of the feasibility study will be judged quantitatively relating to the recruitment rate, adherence to intervention requirements, and collection of outcome data. A traffic light system will be used (see Table 3). If all criteria are green, we will proceed to a full-scale multisite trial. If one or more are amber, we will propose adaptations to address limitations. If one or more criteria are red, we will discuss with our study steering committee what further adaptations could be made, and whether the trial is feasible.

**Table 3:**
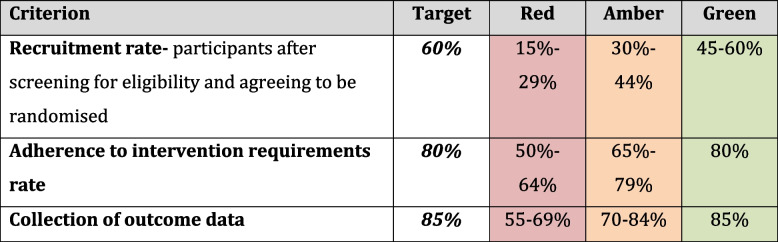
Success criteria to determine move to full-scale trial

Acceptability of trial processes and use of isCGM in women in both trial arms and clinical HCPs will be evaluated through the qualitative study. Criteria for progression to full-scale multisite trial will be participants and HCPs expressing acceptability of trial processes including recruitment, randomisation, outcome measures and follow-up. Acceptability of the isCGM (intervention arm) and masked device (control arm) will be explored during interviews. Barriers to acceptability will be addressed through adaptations to the trial protocol.

## Discussion

Prevalence of GDM is increasing worldwide and confers risk of adverse perinatal outcomes. Reducing time in hyperglycaemia reduces risk of these outcomes. CGM offers an opportunity to provide an improved picture of blood glucose fluctuations to enable changes to clinical management and lifestyle recommendations compared with current SMBG. However, despite evidence of benefit in women with type 1 diabetes mellitus, there is little high-quality evidence from RCTs to understand its potential efficacy in women with GDM, with studies limited by small sample sizes, and short-term use of the devices. The James Lind Alliance have identified using diabetes health technology to improve pregnancy, birth and mother and child health outcomes as the number one current research priority in this group of patients [47].

The RECOGNISE multi-site feasibility study aims to address questions relating to the delivery of a large-scale randomised controlled trial of isCGM compared with normal care (SMBG), including recruitment rates, attrition, adherence to device requirements, potential effect sizes for the primary outcome, and data capture for health economic analyses. It will also explore acceptability of trial participation and use of isCGM in women with GDM, as well as the views of their clinical team. We will use methods designed to engage women from underserved communities, including BAME women, and those that do not speak English as a first language. isCGM could offer a timely, easy to engage with intervention, to improve glycaemic control, reducing adverse pregnancy, birth, and long-term health outcomes for mother and child. The data provided by the RECOGNISE feasibility study will inform development of a large-scale, multi-site randomised controlled trial to assess the effectiveness of CGM for clinical, psychosocial and and health economic outcomes in women with GDM.

### Dissemination policy

We will disseminate our work by publishing in a high impact factor peer reviewed journals and at relevant national and international conferences. A lay summary of the results will be co-produced with our patient partner group. For peer reviewed publications ICJME guidelines [48] will be used to determine authorship.

### Sponsor

North Bristol NHS Trust is the Sponsor for this study. The Sponsor supported the design and will support execution of this study, but will have no role in the analyses or interpretation of data.

Contact:

Sponsor, North Bristol NHS Trust

Learning and Research,

Southmead Hospital,

Bristol, BS10 5NB.

Email: researchsponsor@nbt.nhs.uk.

### Trial management committees and monitoring

The study will be monitored in accordance with the Sponsor’s (NBT) Standard Operating Procedure. The organisational structure and responsibilities are documented in Additional file 1. All documents will be made available on request for monitoring and audit by NBT, the HRA or other licensed bodies. The monitoring plan will be developed and agreed by the Sponsor and will be conducted by Sponsor representatives.

There is a trial management group comprising the trial manager, CI, PI, theme leads, and research delivery staff who will meet at least every 8 weeks to monitor site procedures. There is a Study Steering Committee made up of independent experts and a patient representative who oversee implementation and will meet 3-monthly to monitor progress of the trial. The decision to stop the trial prematurely will be made by the Study Steering Committee and the Sponsor if deemed appropriate on review of any Serious Adverse Events. Due to the design of this study (feasibility), there is no data monitoring committee, and data monitoring will instead be conducted by the Study Steering Committee.

## Supplementary Information

**Additional file 1. **RECOGNISE: Organisational structure and responsibilities.

## Data Availability

No data have been generated or analysed for this protocol. Direct access to documentation and materials will be granted to authorised representatives from the Sponsor, host institution and the regulatory authorities to permit trial-related monitoring, audits, and inspections—in line with participant consent. Availability of the study protocol and data generated will be specified in the data management plan which will be developed before commencement of the study, in line with sponsor and funder (NIHR) guidance.

## Abbreviations

isCGM: Intermittently scanned continuous glucose monitoring
CGM: Continuous glucose monitoring
CRF: Case Report Form
GDM: Gestational diabetes mellitus
HbA1C: Haemoglobin A1c
HCP: Healthcare professional
HRA: Health Research Authority
LGA: Large for gestational age
NBT: North Bristol NHS Trust
NHS: National Health Service
NICE: National institute of Health and Care Excellence
NICU: Neonatal intensive care unit
OASI: Obstetric and sphincter injury
PPAQ: Pregnancy physical activity questionnaire
PPG: Patient Partner Group
PSS: Personal social services
QuInteT: Qualitative Research Integrated in Trials
REC: Research Ethics Committee
RECOGNISE: TaRgeted intermittEnt gluCose mOnitoring for the management of GestatioNal dIabeteS mellitus
RCT: Randomised controlled trial
SAE: Serious adverse event
SFT: Somerset Foundation Trust
SGA: Small for gestational age
SMBG: Self-monitoring of blood glucose
T2DM: Type 2 diabetes mellitus
TFA: Theoretical Framework of Acceptability
UK: United Kingdom
