# Supplementary material for: Protocol for a multi-site randomised controlled feasibility study investigating intermittently scanned blood continuous glucose monitoring use for gestational diabetes: the RECOGNISE study
Source: Pilot Feasibility Stud. 2023 Jul 11;9:120. doi: 10.1186/s40814-023-01341-y (PMC10334522; doi:10.1186/s40814-023-01341-y)
Supplement: Supplementary file 1 — Additional file 1. RECOGNISE: Organisational structure and responsibilities. [file 40814_2023_1341_MOESM1_ESM.docx]

**RECOGNISE: Organisational structure and responsibilities**

**Principal Investigators**

- Design and conduct of RECOGNISE
- Preparation of protocol and revisions
- Preparation of standard operating procedures
- Organising steering committee meetings
- Publication of study reports
- Members of TMG (Trial Management Group)
- Responsible for identification, recruitment, data collection and completion of Case Report Forms (CRFs), along with follow up of study patients and adherence to study protocol

**Study steering committee**

- Agreement of final protocol
- Periodic review of progress of study and if necessary agreeing changes to the protocol and/or standard operating procedures to facilitate the smooth running of the study
- Advice for lead investigators

**Trial Management Group (TMG)**

- Study planning
- Leadership of work packages
- Assistance with ethics committee applications
- Regular review of progress of study and if necessary agreeing changes to the protocol and/or standard operating procedures to facilitate the smooth running of the study
- Co-production of study summary report/s

**Sponsor/ Trial Manager**

- Management of study database
- Data quality assurance
- Serious Adverse Event (SAE) monitoring
- Organisation of steering committee meetings
- Budget administration and contractual issues with individual centres
- Responsible for trial master file
- Randomisation

**Research delivery team**

- Responsible for identification, recruitment, data collection and completion of CRFs, along with follow up of study patients and adherence to study protocol.
- Screening and informed consent
- Study specific clinical procedures
- Data collection and completion of CRFs

**Patient Partner Group**

- Contribution to study design
- Review and final approval of patient-facing study documentation
- Co-production of study summary report/s
